# Supplementary figures and images for: A TGFB2/TNF-induced in vitro model of proliferative vitreoretinopathy (PVR) using ARPE-19 cells confirms nicotinamide as an inhibitor of EMT and VEGFA secretion
Source: PLoS One. 2026 Jan 13;21(1):e0340614. doi: 10.1371/journal.pone.0340614 (PMC12798965; doi:10.1371/journal.pone.0340614)

**Supplementary Figure SF3**

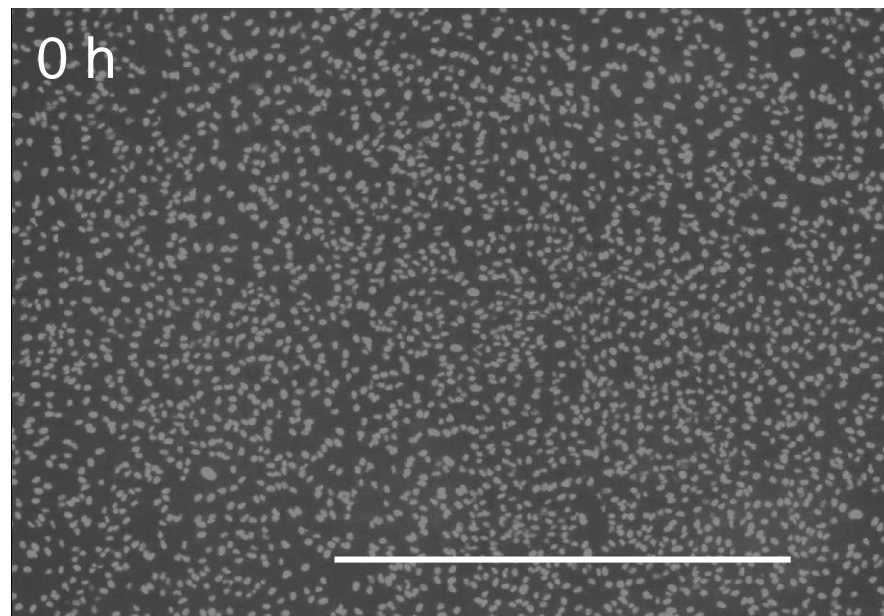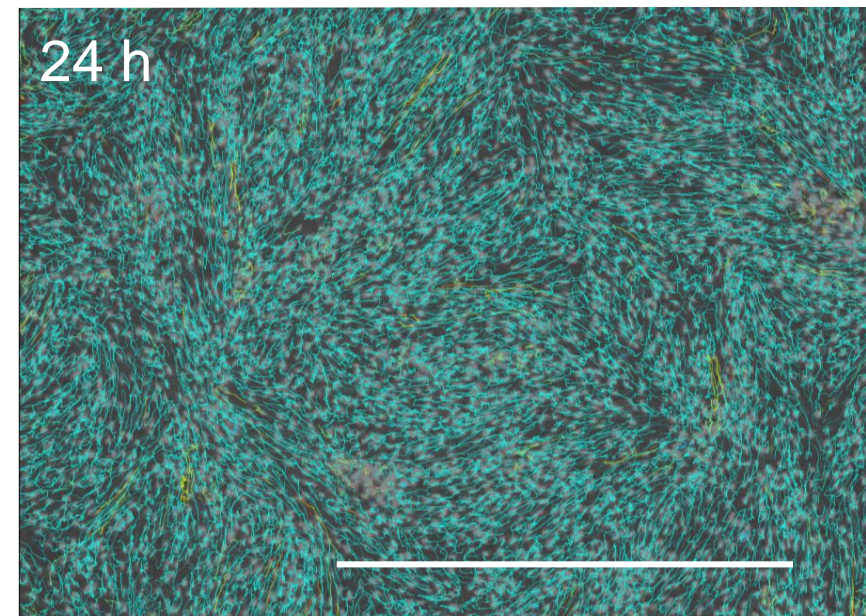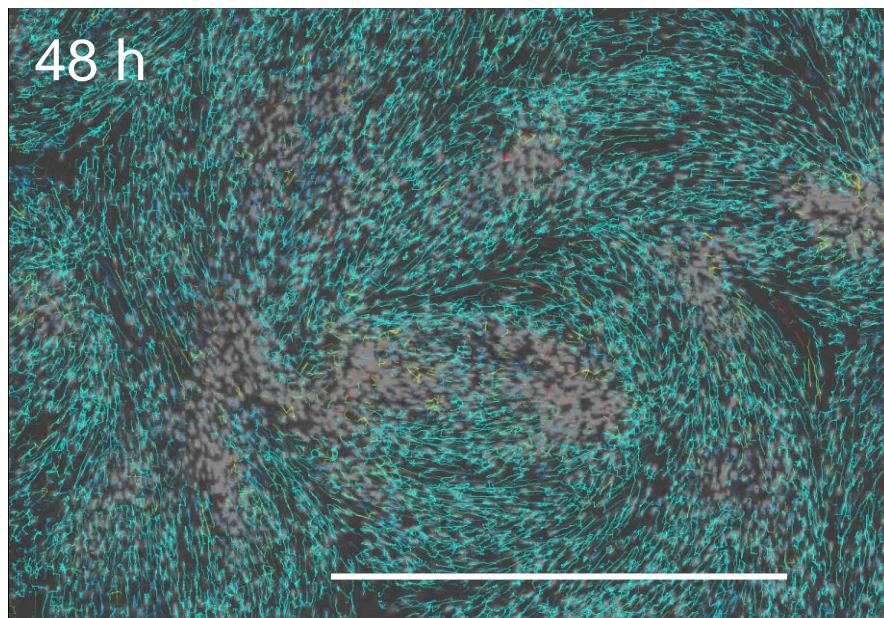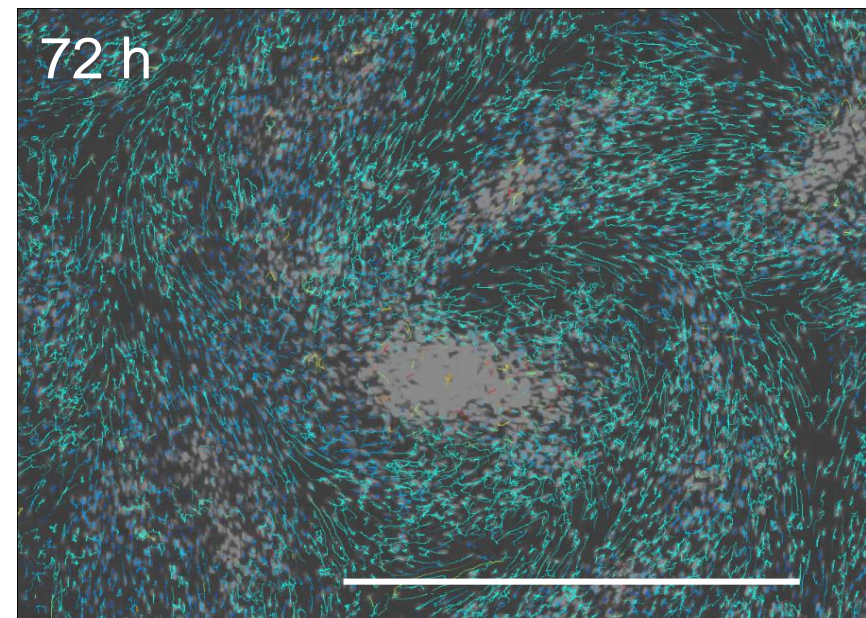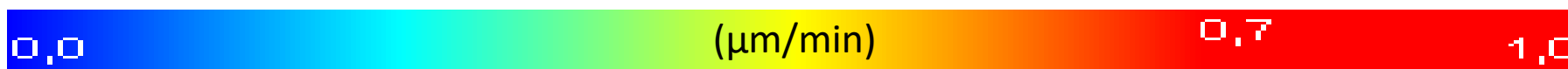

Supplement: S3 Fig — Color-coded migration velocity maps of ARPE-19 cells exposed to TNT for 24, 48, and 72 hours. Nuclear trajectories were tracked 10 h backwards in time using live-cell imaging and analyzed with the StarDist/TrackMate plugin in FIJI. Hoechst-stained nuclei are shown in grey. The scale bar represents 1000 µm. (PDF) [file pone.0340614.s003.pdf]

Supplementary Figure SF5

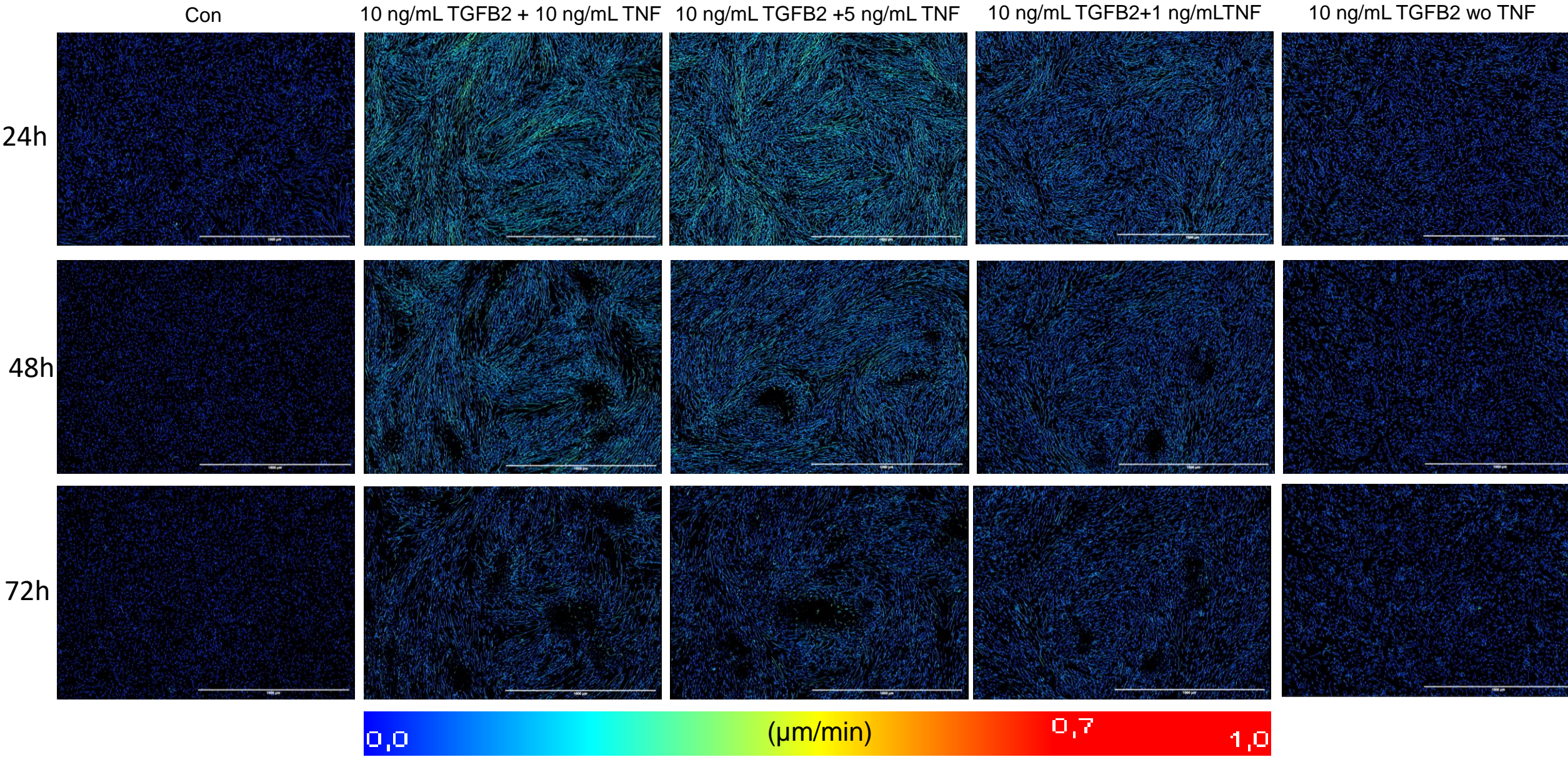

Supplement: S5 Fig — Confluent ARPE-19 monolayers were treated with control medium (Con), 10 ng/mL TGFB2 + 10 ng/mL TNF, 10 ng/mL TGFB2 + 5 ng/mL TNF, 10 ng/mL TGFB2 + 1 ng/mL TNF, or 10 ng/mL TGFB2 alone and subjected to live-cell imaging. Hoechst-stained nuclei were tracked with StarDist/TrackMat plugin in FIJI, and trajectories were reconstructed for a 10-h tracking window ending at the indicated 24 h, 48 h, and 72 h time points. Tracks are color-coded for instantaneous migration velocity according to the heat map (0–1.0 µm/min). Scale bar: 1000 µm. (PDF) [file pone.0340614.s005.pdf]

Supplementary Figure SF8

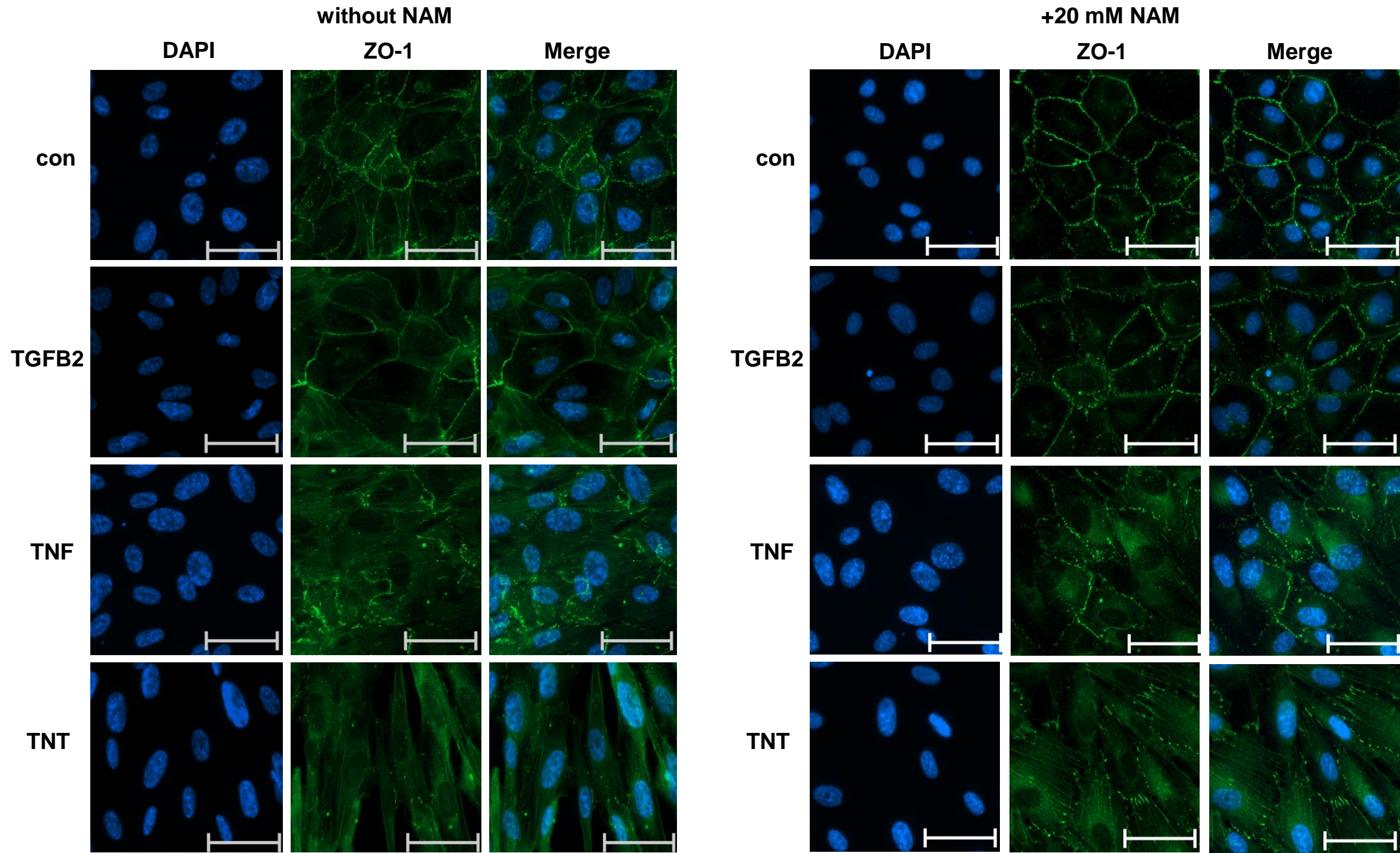

Supplement: S8 Fig — ARPE-19 monolayers were exposed for 5 days to control medium (con), TGFB2, TNF, or the combination of TGFB2 + TNF (TNT) in the absence (left panel, “without NAM”) or presence (right panel, “with NAM”) of 20 mM nicotinamide (NAM). Cells were fixed and stained for ZO-1 (green) and counterstained with DAPI (blue); merged images are shown in the right column of each panel. TNT corresponds to 10 ng/mL TGFB2 + 5 ng/mL TNF. Images are representative of three independent experiments. Scale bar: 50 µm. (PDF) [file pone.0340614.s008.pdf]
