# Supplementary material for: A TGFB2/TNF-induced in vitro model of proliferative vitreoretinopathy (PVR) using ARPE-19 cells confirms nicotinamide as an inhibitor of EMT and VEGFA secretion
Source: PLoS One. 2026 Jan 13;21(1):e0340614. doi: 10.1371/journal.pone.0340614 (PMC12798965; doi:10.1371/journal.pone.0340614)

# Supplementary Figure SF4

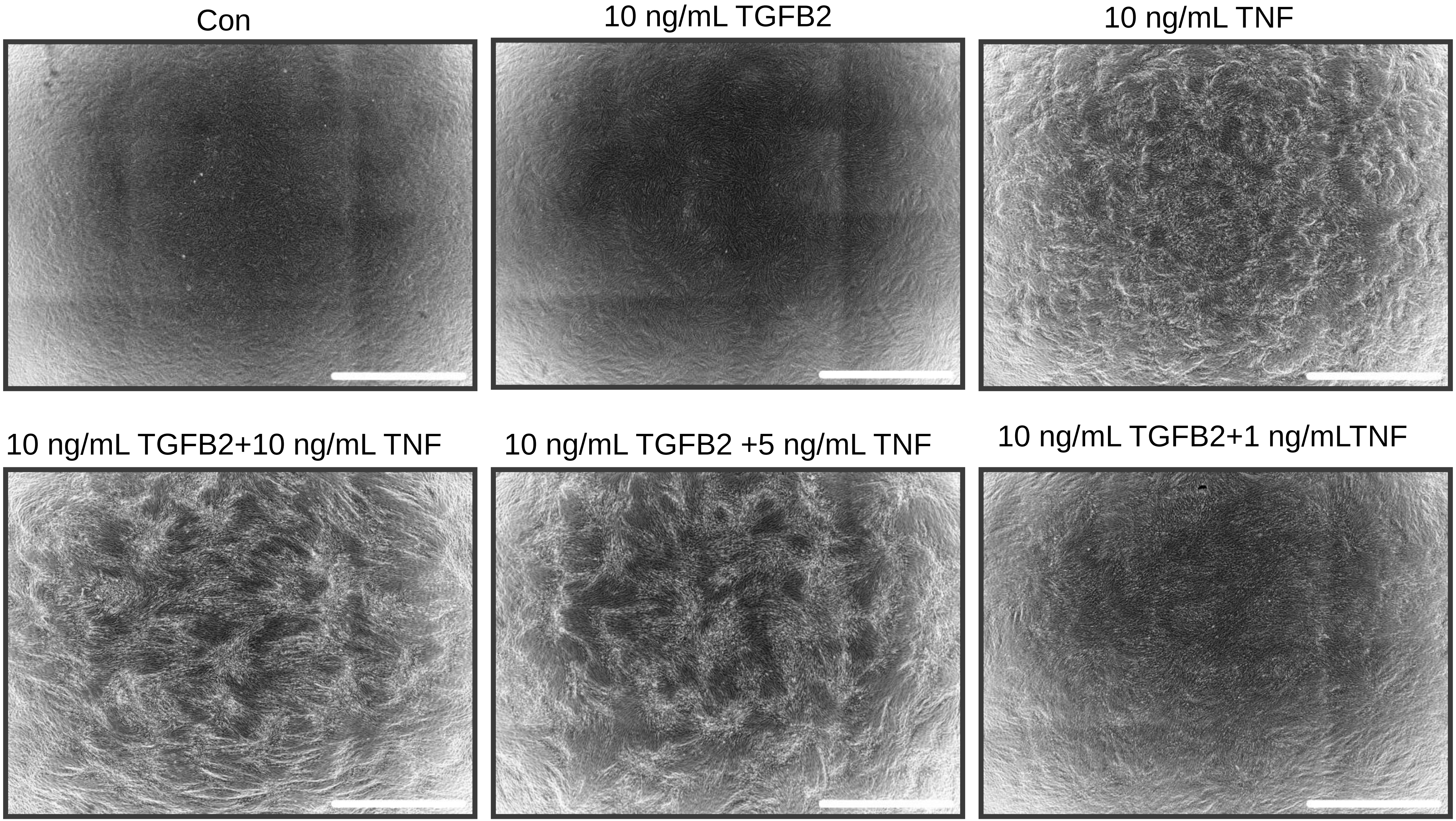

Supplement: S4 Fig — Representative phase-contrast images of ARPE-19 cells three days after treatment with 10 ng/mL TGFB2 and varying concentrations of TNT (0, 10, 5, and 1 ng/mL TNF in combination with 10 ng/mL TGFB2). The scale bar represents 2000 µm. (PDF) [file pone.0340614.s004.pdf]
