# Supplementary material for: A TGFB2/TNF-induced in vitro model of proliferative vitreoretinopathy (PVR) using ARPE-19 cells confirms nicotinamide as an inhibitor of EMT and VEGFA secretion
Source: PLoS One. 2026 Jan 13;21(1):e0340614. doi: 10.1371/journal.pone.0340614 (PMC12798965; doi:10.1371/journal.pone.0340614)
Supplement: S7 Fig — (A) RAW Western blot image showing FN1 protein used for densitometric quantification presented in Fig 4C. Each lane represents protein lysates from ARPE-19 cells treated under the indicated conditions. (B) RAW Western blot image showing total protein bands used for densitometric quantification presented in Fig 4C. Each lane represents protein lysates from ARPE-19 cells treated under the indicated conditions. (PDF) [file pone.0340614.s007.pdf]

Suplementary Figure SF7A

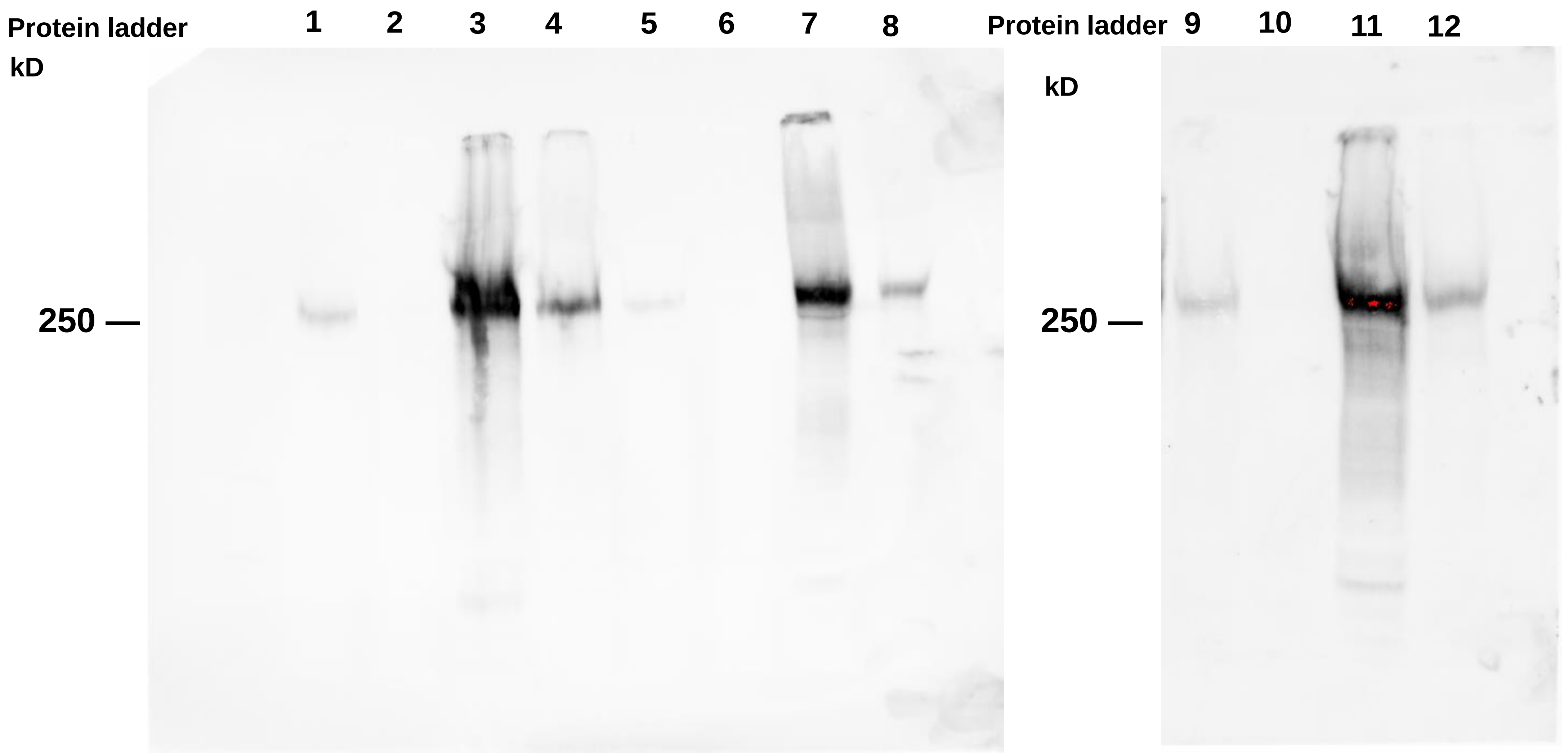

Raw FN1 blot for Fig. 4C.
Lanes 1–4, 5–8, and 9–12 represent three independent biological replicates, each loaded as follows: 1, 5, 9: Con; 2, 6, 10: Con + NAM; 3, 7, 11: TNT; 4, 8, 12: TNT + NAM. The image was captured with a ChemiDoc MP Imaging System (Bio-Rad).

Suplementary Figure SF7B

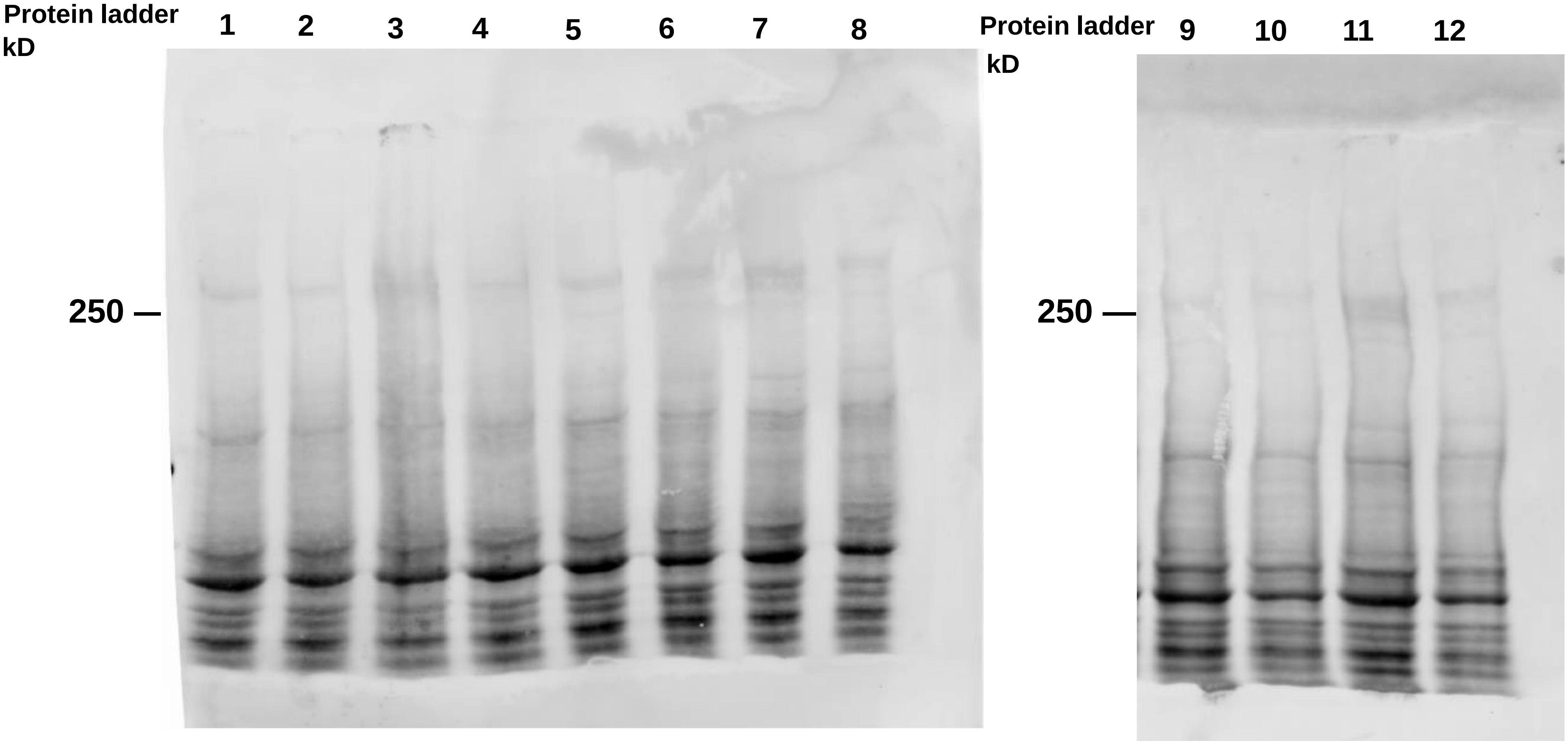

Raw total protein blot for FN1 normalization Fig. 4C.
Lanes 1–4, 5–8, and 9–12 represent three independent biological replicates, each loaded as follows: 1, 5, 9: Con; 2, 6, 10: Con + NAM; 3, 7, 11: TNT; 4, 8, 12: TNT + NAM. The image was captured with a ChemiDoc MP Imaging System (Bio-Rad).
