# Supplementary material for: A TGFB2/TNF-induced in vitro model of proliferative vitreoretinopathy (PVR) using ARPE-19 cells confirms nicotinamide as an inhibitor of EMT and VEGFA secretion
Source: PLoS One. 2026 Jan 13;21(1):e0340614. doi: 10.1371/journal.pone.0340614 (PMC12798965; doi:10.1371/journal.pone.0340614)
Supplement: Attachment — Submitted filename: Comments to the authors.pdf [file pone.0340614.s015.pdf]

Comments to the authors

Authors have investigated PVR related EMT using ARPE-19 cell line and TGF-β and TNF-α induced EMT model. They have also tested nicotinamide potential to prevent EMT.

Abstract

-TNT is confusing and the text does not tell what it means. Please revise and make easy for reader to get meaning already from abstract.

Material and methods

- Need to fulfill reagent information e.g. supplier country information

- western blot line 177, complete cells were treated.

-add species of antibodies e.g. FN1.

- marking of the sample amount is confusing, lines 208-209 . Total sample 9? Please clarify into text.

- Add also concentration information to TGF-β and TNF-α.

- Why ANOVA was used even the sample amount was so small?

Results

- Make figures bigger and more visible.

- Supplementary video 1. Why TNT cell layer is damaged at the end? Is concentrations too high? In which timepoint other measurements have done? Are EMT markers formed due to death cells? Does video present migration? Basicly, when treat cells too high concentrations they always detach from cell plate well. Now figures and supplementary files are separated from text at the end, thus hard to follow exactly.

- Supplementary video SV2. Why are the nuclei accumulated almost on one side of the cell plate well? What it mean and what is the conclusion of it.

- Is sample amount three? Refering supplementary Table related to Fig 6. It is quite small sample amount. Is there done only on repetition with three samples? In figure legend, there should be mentione the sample amount (n).

-It would be interesting to see some comparing epithelial markers changes (e.g. occluding, E-cadherin, or ZO-1) to mesenchymal markers increase (e.g. α.SMA, N-cadherin, fibronectin, vimentin) after all treatments compared to control either in protein or gene expression levels.

- Fig 2, Why nuclei tend to be packed as densely. What does it mean? Make Fig 2 more visible. Now it is quite cloudy for interpret.

- Fig 2 B. Why is velocity scale (μm/min) with colours presented at the below of the figure? The figure does not really present any movement and the velocity at the below does not tell anything. Even colours presented in the velocity scale are not marked or presented in the figure. Please mark or explain velocity meaning or delete it under the figure and from legend. White dots represent aggregates. That should be shown also some concretic way. Now it is only showed by white dots which really not be as result. Formed aggregates should be proven to be formed at that site. It can just also be empty area in cell plate well for example due to death and detach cells. Should be proven to be aggregate. Otherwise need to be deleted from the figure and legend.

-Line 317. “lower TNF concentrations are associated with reduced migration velocity and shorter migration duration”. Are cells really migrated at higher concentrations or just death due to too high concentration and detach from the well bottom? Detach in cytotoxic concentrations is typical for ARPE-19 cells. Migration should be indicated also other way for example by cell wound assay or by invasion assay. Not only by imaging cells in well during treatment. It is not enough if not cytotoxicity or cell viability assays are not shown. Can lead to misinterpretation.

-There should also be presented cytotoxicity or cell viability assaya. For example NAM results need it but also TGF-β, TNF-α, and TNT would be more strong results if those are presented.

Discussion

-There could be more references to cited. For example lines 432-462. There are reported results but they are not reflected at all for the previous literature/research.

-References are not showed/used when needed for example paragraph starting from line 471. Also need to go similar kind of things through manuscript. That paragraph inform background information not shown in this manuscript and reference to this knowledge is needed. Also conclusion is not line with results. In this manuscript is not presented results related NF-kB and MAPK or smad. There can not be concluded to affect these pathways and should mention may all refere little bit different way to TGF and TNF.

-Not much used references and should be more. Also some part of the text was without reference when it was needed (scientific information that sounds familiar for authors but need to be presented with reference when not discusse the results of this manuscript.
