# Supplementary material for: A TGFB2/TNF-induced in vitro model of proliferative vitreoretinopathy (PVR) using ARPE-19 cells confirms nicotinamide as an inhibitor of EMT and VEGFA secretion
Source: PLoS One. 2026 Jan 13;21(1):e0340614. doi: 10.1371/journal.pone.0340614 (PMC12798965; doi:10.1371/journal.pone.0340614)
Supplement: Attachment — Submitted filename: Letter to the reviwer.docx [file pone.0340614.s016.docx]

**Forschungslabor Experimentelle Augenheilkunde**

**Dr. rer. nat Heiko Fuchs, Laborleiter**

OE6120

Heiko Fuchs

Telefon:	0511 532-7246

Fax:	0511 532-7276

fuchs.heiko@mh-hannover.de

Carl-Neuberg-Straße 1

30625 Hannover

Telefon: 0511 532-0

www.mh-hannover.de

**PLOS One**

Dear Prof. Xiaodong Chen,

We would like to thank you for the constructive feedback on our manuscript **“A TGFB2/TNF-Induced *in vitro* model of Proliferative Vitreoretinopathy (PVR) using ARPE-19 cells confirms Nicotinamide as an inhibitor of EMT and VEGFA secretion”**.

We carefully addressed all comments raised during the review process. Below, we provide a point-by-point response highlighting the changes implemented in the revised version of the manuscript.

**Reviewer’s comment 1:**
There is no internal control in the western blot shown in Figure 1 and 4.

**Response:**
Thank you for pointing this out. Instead of using a conventional loading control such as β-actin or GAPDH, in this study we normalized all western blot data to total protein levels, which were detected by stain-free imaging. This technique directly visualizes the total protein in each lane based on the intrinsic fluorescence of tryptophan residues, providing a reliable and unbiased reference for normalization. Representative stain-free images are now included for reference.

Unfortunately, this was not clearly described in the submitted version of the manuscript. We have therefore revised the Methods section to explicitly state:

*“After transfer, total protein bands were visualized using the ‘Stain-Free blot’ option of the ChemiDoc™ Imaging System following 45 s UV activation and automatic exposure acquisition.”*

*“Protein band intensities were normalized to the corresponding total protein signal instead of a housekeeping protein.”*

For reasons of space and figure clarity, these stain-free blots are not shown in the main figures but the corresponding raw stain-free total protein blots have been included in the Supplementary Figures SF3–SF6 to ensure full transparency.

In addition, as requested by the Editor, all raw data underlying the presented figures have been provided as a separate supplementary file Supplementary Table ST1 -raw data.xlsx.

**Reviewer’s comment 2:**
Some data about mechanisms related to the induction of PVR by TGFB2/TNF are lacking in the text.

**Response:**
We appreciate this insightful comment. In the present study, our main objective was to establish a reliable model of PVR induction by TGFβ2/TNF, and we therefore focused primarily on characterizing the phenotypic and functional changes in this model. We did not perform detailed mechanistic experiments at this stage. However, we have revised the Discussion to better contextualize our findings within known signaling mechanisms. Specifically, we now emphasize that TGFβ2 predominantly signals via Smad2/3 phosphorylation to drive EMT and extracellular matrix production, whereas TNF activates NF-κB and MAPK pathways to enhance inflammation and amplify fibrotic responses. Their combined stimulation therefore creates a synergistic pro-fibrotic environment that models central mechanisms thought to contribute to PVR pathogenesis. We further highlight that nicotinamide attenuates both TGFβ2/Smad- and TNF/NF-κB–associated responses in our system, suggesting a dual inhibitory action across multiple signaling axes.

The revised text is included in the Discussion, stating:

*“Mechanistically, TGFβ2 and TNF activate distinct but converging signaling cascades that jointly drive EMT and fibrosis in RPE cells. TGFβ2 primarily signals via Smad2/3 phosphorylation, promoting transcription of extracellular matrix components and EMT regulators, whereas TNF activates NF-κB and MAPK pathways, enhancing inflammatory gene expression and amplifying fibrotic responses. Their combined stimulation therefore provides a synergistic pro-fibrotic environment that reflects key aspects of cytokine-driven mechanisms implicated in PVR. Our data demonstrate that nicotinamide effectively attenuates both TGFβ2/Smad- and TNF/NF-κB–associated responses, suggesting that its inhibitory effect extends across multiple signaling axes. This dual blockade may underlie the robust suppression of EMT markers, contractility, and VEGFA secretion observed in our model.”*

We believe that these revisions have significantly improved the clarity and completeness of our manuscript. We thank the reviewer for their valuable input and hope that the revised version meets the journal’s standards for publication.

Sincerely,

Heiko Fuchs
on behalf of all co-authors

Department of Ophthalmology
Hannover Medical School
Carl-Neuberg-Straße 1, 30625 Hannover, Germany
Email: fuchs.heiko@mh-hannover.de
