# Supplementary material for: A TGFB2/TNF-induced in vitro model of proliferative vitreoretinopathy (PVR) using ARPE-19 cells confirms nicotinamide as an inhibitor of EMT and VEGFA secretion
Source: PLoS One. 2026 Jan 13;21(1):e0340614. doi: 10.1371/journal.pone.0340614 (PMC12798965; doi:10.1371/journal.pone.0340614)
Supplement: Attachment — Submitted filename: Letter to the reviewersHF_211125.docx [file pone.0340614.s017.docx]

**Forschungslabor Experimentelle Augenheilkunde**

**Dr. rer. nat Heiko Fuchs, Laborleiter**

OE6120

Heiko Fuchs

Telefon:	0511 532-7246

Fax:	0511 532-7276

fuchs.heiko@mh-hannover.de

Carl-Neuberg-Straße 1

30625 Hannover

Telefon: 0511 532-0

www.mh-hannover.de

Dear reviewers,

21. Nov. 2025

First, we thank the reviewers for taking the time to read and review our manuscript. We have improved the manuscript based on their comments and suggestions and would like to address their points in detail below.

**Responses to Reviewer #1 comments**

**Reviewer #1 - additional comment (stain-free total protein blots):**

“The author mentioned in methods section to explicitly state: “After transfer, total protein bands were visualized using the ‘Stain-Free blot’ option of the ChemiDoc™ Imaging System following 45 s UV activation and automatic exposure acquisition.” However, the corresponding raw stain-free total protein blots have not been included in the Supplementary Figures SF3-SF6. Please the author check and add or label raw stain-free total in the Supplementary Figures SF3-SF6.”

**Response:**
We thank the reviewer for carefully checking this point. In the original submission, the labelling and numbering of the supplementary figures were indeed not fully consistent with the description in the text, and the stain-free total protein images were not clearly identifiable.

In the revised version, we have corrected this and now provide, for each western blot experiment, both the raw immunoblot and the corresponding stain-free total protein blot as separate panels, with explicit labelling and highlighting the numbers of biological replicates. Specifically:

- **Supplementary Figure SF1A-B**: COL1A1 immunoblot and matching stain-free total protein blot for Figure 1C
- **Supplementary Figure SF2A-B**: FN1 immunoblot and matching stain-free total protein blot for Figure 1C
- **Supplementary Figure SF6A-B**: COL1A1 immunoblot and matching stain-free total protein blot for Figure 4C
- **Supplementary Figure SF7A-B**: FN1 immunoblot and matching stain-free total protein blot for Figure 4C

We have also updated the references to the supplementary material in the main text and Methods section so that the numbering now correctly points to these stain-free total protein controls. All corresponding densitometric data are included in **Supplementary Table ST1 (Raw data).**

We hope that this revised presentation fully addresses the reviewer’s concern regarding the availability and labelling of the stain-free total protein blots.

**Responses to Reviewer #2 comments**

**Reviewer Comment 1 (Abstract):**

“TNT is confusing and the text does not tell what it means. Please revise and make easy for reader to get meaning already from abstract.”

**Response:**
We thank the reviewer for this helpful remark. We agree that the abbreviation “TNT” should be introduced as clearly as possible in the abstract. We have therefore revised the relevant sentence to explicitly define the combined cytokine stimulus, which now reads as follows:
“Here, we establish a cytokine-driven *in vitro* PVR model by co-stimulating ARPE-19 cells with transforming growth factor beta 2 (TGFB2) and tumor necrosis factor-alpha (TNF); this combined TGFB2/TNF stimulus is hereafter referred to as ‘TNT’.”
We hope that this revised wording makes the meaning of the abbreviation immediately clear to the reader.

**Reviewer Comment 2 (Materials and Methods – reagents):**

“Need to fulfill reagent information e.g. supplier country information.”

**Response:**
We thank the reviewer for this comment and fully agree that transparent reagent information is essential for reproducibility. In the Materials and Methods section, we already provide the catalogue numbers (and, where applicable, the supplier names) for all reagents used. As these catalogue numbers are globally unique and independent of the supplier’s local branch or country, they allow readers worldwide to obtain the identical reagents without ambiguity. We therefore did not further specify supplier country information, as we consider the current level of detail sufficient to ensure reproducibility.

**Reviewer Comment 3 (Western blot):**

“Western blot line 177, complete cells were treated.”

**Response:**
We thank the reviewer for this helpful remark and agree that the original wording could be misinterpreted. We have therefore clarified the description in the Methods section to make explicit that intact ARPE-19 cells were treated prior to lysis. The revised sentence now states:
“After 24 h, cells were treated with TGFB2, TNF, TNT, or control with or without NAM for five days before lysis.” This wording should avoid any ambiguity regarding the treatment of complete cells before protein extraction.

**Reviewer Comment 4 (Antibodies):**

“Add species of antibodies e.g. FN1.”

We thank the reviewer for this helpful suggestion. We have revised the Methods section to explicitly state the host species of the primary antibodies. The respective sentence now reads:
“Membranes were blocked with 5% milk powder (Carl Roth #T145.2) in 1× Tris-buffered saline (TBS) at room temperature (RT) for 1 h, followed by overnight incubation at 4°C with a 1:1000 dilution of Fibronectin 1 (FN1) rabbit mAB (Cell Signaling #26836S) and COL1A1 rabbit mAB (Cell Signaling #72026S).”

**Reviewer Comment 5 (Sample amount):**

“Marking of the sample amount is confusing, lines 208–209. Total sample 9? Please clarify into text.”

**Response:**
We thank the reviewer for this comment. In the Materials and Methods section, we specify the replicate structure as follows:“Each condition was analyzed in three technical replicates across three biological replicates.”This indicates that three independent biological experiments were performed (n = 3), with each biological replicate measured in triplicate (technical replicates). To make the total number of biological replicates more immediately transparent for the reader, we now additionally state the number of biological replicates (n) in the respective figure legends and list them in detail in Supplementary Table ST1 (“Raw data”).

**Reviewer Comment 6 (Concentrations)** “Add also concentration information to TGF-β and TNF-α.”

**Response:**
We thank the reviewer for this helpful suggestion. We have already clarified the cytokine concentrations in the Materials and Methods section. The text states that ARPE-19 cells were treated with **10 ng/mL TGFB2** and **5 ng/mL TNF**, unless otherwise indicated (e.g.SF 3+4).

**Reviewer Comment 7 (Statistics):**

“Why ANOVA was used even the sample amount was so small?”

**Response:**
We thank the reviewer for this comment. In our experiments we routinely compared 4–8 different conditions (control, TGFB2, TNF, TNT, with or without NAM). In such settings, a series of pairwise t-tests would markedly increase the risk of type I error, whereas one-way ANOVA is specifically designed to test for differences across multiple groups within a single model. Although the number of biological replicates per group is relatively small (n ≥ 3), this approach is widely used and appropriate for exploratory *in vitro* studies with multiple treatment conditions.

**Reviewer Comment 8 (Results – Figures):**

“Make figures bigger and more visible.”

**Response:**
We thank the reviewer for this helpful suggestion. In the revised manuscript, we have increased the sizes of Figure 2 and Figure 5 to improve visibility and interpretation. We would also like to note that the figures displayed in the review PDF generated by the PLOS ONE submission system are subject to automatic downscaling and compression, which can reduce their apparent resolution. The original figure files that we uploaded are high-resolution images, and in the final typeset version of the article, the publisher will use these full-resolution files so that readers can zoom in without a substantial loss of image quality.

**Reviewer Comment 9 (Supplementary Video 1):**

“Supplementary video 1. Why TNT cell layer is damaged at the end? Is concentrations too high? In which timepoint other measurements have done? Are EMT markers formed due to death cells? Does video present migration? Basicly, when treat cells too high concentrations they always detach from cell plate well.”

**Response:**
We thank the reviewer for these questions and for the careful inspection of Supplementary Video 1, and we apologize that an important piece of information was missing in the original legend. In the revised manuscript, we have now clarified that the video shows phase-contrast live-cell imaging of control- and TNT-treated ARPE-19 cells after 72 h of prior exposure to the respective conditions. From this time point onward, TNT-treated cells increasingly migrate and cluster into multicellular, multilayered aggregates with pronounced contractile behavior. The legend of Supplementary Video 1 has been updated accordingly.

The apparent “damage” of the TNT cell layer at later time points is therefore not due to acute cytotoxicity from excessively high cytokine concentrations, but reflects mechanically driven detachment of the monolayer caused by these contractile aggregates. As the aggregates pull on the surrounding sheet, the cell layer folds and partially lifts off the substrate, which is consistent with the membrane-like contraction described in proliferative vitreoretinopathy (PVR). In pilot experiments using propidium iodide (PI) staining, we did not observe an increased frequency of PI-positive nuclei under TNT conditions compared with controls, which is why we did not further pursue this readout in the final experimental series (data not shown). During the imaging period, we do not observe massive loss of cells or widespread nuclear fragmentation that would be expected with extensive cell death.

The cytokine concentrations used in our model (10 ng/mL TGFB2 and 5 ng/mL TNF, unless otherwise indicated) are within the range commonly applied in *in vitro* PVR/EMT models and were chosen because they robustly induce fibrotic remodeling without causing global cell loss. EMT and fibrotic markers (FN1, COL1A1, ZO-1 and F-actin reorganization) and VEGFA secretion were quantified at defined end points after TNT exposure (e.g. day 5), as specified in the Methods, i.e. at time points when TNT-treated cultures still consist of viable, contracting cell layers and aggregates. Thus, the increased EMT marker levels reflect active fibrotic remodeling rather than artefacts arising from dying cells.

Finally, Supplementary Video 1 indeed documents TNT-induced migration: cells undergo collective migration towards emerging foci, followed by three-dimensional piling-up into contractile aggregates. This behavior is further quantified and visualized in the main figures and supplementary figures, and the video is intended as a qualitative illustration of these TNT-induced migration and aggregation dynamics.

**Reviewer Comment 10 (Supplementary Video 2):**

“Why are the nuclei accumulated almost on one side of the cell plate well? What does it mean and what is the conclusion of it.”

**Response:**
We thank the reviewer for this observation. Supplementary Video 2 was recorded with a 4× objective in a fixed field of view (FOV) within a 24-well plate. At this magnification, the camera captures only a small fraction of the total growth surface, not the entire well. The apparent accumulation of nuclei “on one side of the well” therefore reflects local aggregate formation within this limited FOV rather than a global asymmetry of the whole well.

In replicate recordings, multicellular aggregates formed at multiple locations across the well. The lateral accumulation seen in the presented clip is thus a stochastic local event and illustrates the collective migration and clustering behavior induced under TNT conditions, which is consistent with the PVR-like phenotype we aim to model.

**Reviewer Comment 11 (Sample size):**

“Is sample amount three? Refering supplementary Table related to Fig 6. It is quite small sample amount. Is there done only on repetition with three samples? In figure legend, there should be mentione the sample amount (n).”

**Response:**

We thank the reviewer for this comment. For the experiments related to Figure 6 (and all other quantitative assays in this study), we performed **three to five independent biological experiments**. Thus, the data do not originate from a single experiment with three wells, but from three separate experimental runs. We now explicitly state the number of biological replicates (n) in the corresponding figure legends and in Supplementary Table ST1 (“Raw data”), so that the sample size is immediately transparent to the reader.

**Reviewer Comment 12 (Epithelial vs. mesenchymal markers):**

“It would be interesting to see some comparing epithelial markers changes (e.g. occluding, E-cadherin, or ZO-1) to mesenchymal markers increase (e.g. α.SMA, N-cadherin, fibronectin, vimentin) after all treatments compared to control either in protein or gene expression levels.”

**Response:**
We appreciate this thoughtful suggestion. Our a priori aim in the present study was to characterize fibrotic remodelling rather than to comprehensively stage EMT, which is why we focused on mesenchymal/fibrotic readouts such as FN1, COL1A1, and F-actin stress fibres that directly report matrix production and contractile reorganization relevant to PVR. We did not perform a broader panel of mesenchymal markers (e.g. α-SMA, N-cadherin, vimentin) or gene expression profiling in this work.

To address the reviewer’s comment, we additionally performed immunofluorescence staining for the epithelial tight-junction marker ZO-1 after five days of treatment. Under our culture conditions, robust junctional ZO-1 staining was only observed in confluent monolayers, which were consistently reached at day 5; therefore, this time point was chosen for epithelial marker analysis. The new data are presented in **Supplementary Figure SF8**. In control cells, ZO-1 shows a continuous junctional belt, whereas TNT treatment markedly disrupts this membrane-associated pattern. Co-treatment with nicotinamide partially preserves junctional ZO-1 staining compared with TNT alone, consistent with the overall EMT-attenuating and anti-fibrotic effect of nicotinamide in our model.

We did not analyse occludin or E-cadherin in this study, but agree that combining a broader epithelial and mesenchymal marker panel at the protein and/or transcript level would be an interesting objective for future work.

**Reviewer Comment 13 (Fig. 2 – visibility & dense nuclei):**

“Why nuclei tend to be packed as densely. What does it mean? Make Fig 2 more visible. Now it is quite cloudy for interpret.”

**Response:**
We thank the reviewer for this comment. As requested, we have enlarged Figure 2 in the revised manuscript to improve visibility. The densely packed appearance of the nuclei is an inherent feature of the TNT condition and reflects the underlying biology of our model: under TNT, ARPE-19 cells migrate and cluster into compact, multilayered aggregates rather than remaining in a simple monolayer. In a single-plane live-cell imaging readout, this three-dimensional piling-up of cells appears as closely apposed or overlapping nuclei within the field of view. Functionally, these multilayered, contractile aggregates are a key hallmark of our *in vitro* PVR system, as they are required to generate the pronounced tissue contraction and membrane-like behavior observed in Supplementary Video 1.

**Reviewer Comment 14 (Fig. 2B – velocity scale & aggregates):**

“Fig 2 B. Why is velocity scale (μm/min) with colours presented at the below of the figure? The figure does not really present any movement and the velocity at the below does not tell anything. Even colours presented in the velocity scale are not marked or presented in the figure. Please mark or explain velocity meaning or delete it under the figure and from legend. White dots represent aggregates. That should be shown also some concretic way. Now it is only showed by white dots which really not be as result. Formed aggregates should be proven to be formed at that site. It can just also be empty area in cell plate well for example due to death and detach cells. Should be proven to be aggregate. Otherwise need to be deleted from the figure and legend.”

**Response:**
We thank the reviewer for these helpful comments on Figure 2B. Regarding the **velocity scale**, Figure 2B displays nuclear migration tracks generated with the TrackMate/StarDist pipeline. The colour of each track segment encodes the **instantaneous nuclear velocity (μm/min)** according to the colour bar shown below the panel. In the original submission, this was not explained clearly enough, which may have given the impression that the colour scale was unrelated to the tracks. In the revised manuscript, we have therefore clarified in the figure legend that **track colour directly corresponds to nuclear velocity according to the scale bar**, and that the trajectories shown represent the **10 h preceding each indicated time point.** This makes the dynamic aspect and the meaning of the velocity scale explicit.

Regarding the **aggregates**, we understand the concern that the previously black areas marked by astericks alone were not sufficiently self-explanatory and, in principle, could be misinterpreted as cell-free or detached areas. In the revised version, we have therefore included SF3, showing color-coded velocity tracks together with Hoechst-stained nuclei in greyscale.

- **Supplementary Figure SF3** shows the same field of view with **Hoechst-stained nuclei highlighted in grey and velocity-coded tracks overlaid**, demonstrating that regions where tracks converge correspond to densely packed, multilayered cell clusters.
- **Supplementary Video SV2** illustrates the formation multicellular aggregates under TNT conditions over time by aquisition of Hoechst-stained nuclei.
- **Supplementary Video SV3** shows the corresponding velocity maps, in which trajectories converge into these aggregates.

Together, these supplementary figures and videos demonstrate that the focal areas in Figure 2B where tracks accumulate and change velocity represent **true multicellular aggregates**, rather than empty regions due to cell death and detachment. We hope that these clarifications and the revised cross-references (Fig. 2B ↔ SF3 ↔ SV2/SV3) make the meaning of the velocity scale and the nature of the aggregates clear and adequately address the reviewer’s concerns.

**Reviewer Comment 15 (TNF dose, migration vs. cytotoxicity):**

“Line 317. ‘lower TNF concentrations are associated with reduced migration velocity and shorter migration duration’. Are cells really migrated at higher concentrations or just death due to too high concentration and detach from the well bottom? Detach in cytotoxic concentrations is typical for ARPE-19 cells. Migration should be indicated also other way for example by cell wound assay or by invasion assay. Not only by imaging cells in well during treatment. It is not enough if not cytotoxicity or cell viability assays are not shown. Can lead to misinterpretation.”

**Response:**

We thank the reviewer for raising these points regarding the interpretation of the TNF dose–response experiments. Our model was deliberately designed to study confluent ARPE-19 monolayers under continuous TNT exposure, in order to mimic cytokine-driven PVR-like behavior of an intact cell sheet rather than isolated cells in a scratch or invasion assay. Migration in this context was assessed by time-resolved nuclear tracking in live-cell imaging sequences using the TrackMate/StarDist pipeline. For each condition, individual nuclear trajectories were reconstructed over defined 10 h windows preceding the 24 h, 48 h, and 72 h time points. At higher TNF concentrations within the TNT context, these tracks show continuous displacement and increased trajectory length/velocity during the analyzed intervals, consistent with collective migration of the confluent cell layer towards emerging aggregates, rather than abrupt disappearance of nuclei that would be expected with acute cytotoxic detachment.

We agree that ARPE-19 cells can detach under truly cytotoxic conditions. For this reason, we chose cytokine concentrations (10 ng/mL TGFB2 and 5–10 ng/mL TNF) within the range commonly used in *in vitro* EMT/PVR models and, during the analyzed tracking windows, did not observe widespread loss of nuclei or massive nuclear fragmentation in the live-cell imaging sequences. As already stated in the manuscript, we initially performed propidium iodide (PI) staining in pilot experiments and did not detect a clear increase in PI-positive nuclei under TNT conditions compared with controls; therefore, we did not pursue this readout further in the final experimental series (data not shown).

We agree that wound-healing or invasion assays can provide complementary information on cell motility. However, such assays inherently require an artificial gap or transwell barrier and were therefore not part of the conceptual design of our confluent-monolayer, continuous-stimulation model. In the revised Discussion, we now explicitly state that our conclusions about migration are based on automated nuclear tracking and aggregate formation in confluent cultures, and that future work could integrate additional assay formats and systematic viability measurements to further dissect motility and survival under different cytokine conditions.

To avoid overinterpretation, we have also slightly rephrased the sentence in the Results to read:

“The tracking data indicate that, within the analyzed 10 h windows, nuclear trajectories are faster and more persistent at higher TNF concentrations, consistent with enhanced collective migration before any detectable detachment.”

We hope that these clarifications and the adjusted wording reduce the risk of misinterpretation and adequately address the reviewer’s concern.

**Responses to Reviewer #3 comments**

**Reviewer #3 – Minor point (a)**

“Although the use of ARPE-19 cells is well-justified for establishing this screening model, the Discussion should more prominently acknowledge the inherent limitations compared to primary RPE cells or iPSC-derived RPE, particularly regarding translational relevance. For example, absence of melanin could affect cytokine responses and as as immortalized cells, ARPE-19 may already exhibit mesenchymal features that could overestimate EMT and therapeutic effects.”

**Response:**
We thank the reviewer for this important comment and fully agree that the limitations of ARPE-19 cells with respect to translational relevance should be explicitly acknowledged. In the revised Discussion, we have strengthened the corresponding paragraph to address these issues in more detail. We now point out those ARPE-19 cells, while widely used and well suited for reproducible high-throughput screening and live-cell imaging, are an immortalized cell line and can exhibit a partial EMT-like or dedifferentiated phenotype at baseline. This may influence cytokine responsiveness and could potentially exaggerate the magnitude of EMT/fibrotic responses and inhibitory effects observed in our model when compared with native or iPSC-derived RPE.

We also more clearly emphasize in the Discussion that the TNT-induced aggregate formation observed in our ARPE-19 model is consistent with previous reports using primary human RPE cells exposed to similar cytokine combinations, which likewise describe multicellular, contractile aggregates under PVR-like conditions. This phenomenological agreement supports the relevance of our model, while its inherent limitations are explicitly acknowledged.

We therefore frame our TNT–ARPE-19 system as a convenient, technically robust first-pass screening platform for anti-fibrotic compounds in the context of PVR, and emphasize that promising candidates such as nicotinamide should ultimately be validated in primary or iPSC-derived RPE models to better assess their translational potential.

**Reviewer #3 – Minor point (b)**

*“While there are space limitations, some controls are very important to show in the main manuscript. Thus, including one representative stain-free Western blot in the main figures (alongside the supplementary data) would enhance transparency and allow readers to better assess loading controls and normalization approaches.”*

**Response:**
We appreciate this helpful suggestion and fully agree that transparent presentation of loading controls is important. In all western blot experiments, we used stain-free total protein imaging for normalization, and the corresponding raw and total protein blots for COL1A1 and FN1 are provided in full in Supplementary Figures SF1–SF2 and SF6–SF7.

We carefully considered adding a stain-free blot to the main figures. However, to faithfully represent a *total* protein control, the entire membrane has to be shown. Including an additional full-length total protein panel alongside the existing COL1A1 and FN1 blots would considerably increase the complexity and size of the main figures, which are already dense. Showing only cropped sections of the stain-free blot, on the other hand, would no longer properly reflect the total lane signal and could be misleading.

For these reasons, we decided to keep the stain-free blots in the Supplementary Figures, where they are presented in an uncropped and fully transparent manner. For clarity, we included the following statement in the Figure legends: “The raw western blot images used for quantification are shown in Supplementary Information (SF1+2)/(SF6–SF7).” We hope that this layout provides an appropriate balance between figure clarity in the main text and detailed documentation of loading controls and normalization strategy in the supplement.

We sincerely thank the reviewers for the constructive comments and the careful evaluation of our work, and we hope that the revised manuscript will now be suitable for publication in PLOS ONE.

Kind regards

Heiko Fuchs

on behalf of all co-authors
